# Prevalence of the colonization of *Helicobacter pylori* among students of the school of dentistry, University of Granada, Spain

**DOI:** 10.4317/medoral.21167

**Published:** 2016-07-31

**Authors:** José Liébana, Violeta García-Casas, Francisco Liébana-Cabanillas, María-Teresa Arias-Moliz

**Affiliations:** 1 MD, PhD, Professor. Department of Microbiology, Schools of Dentistry and Medicine. University of Granada, Spain; 2 MD, BDS, Microbiologist. Department of Microbiology, Schools of Dentistry and Medicine. University of Granada, Spain; 3 PhD, Assistant Professor. Department of Marketing and Market Research, School of Economics and Business Administration; 4 DDS, PhD, Assistant Professor. Department of Microbiology, Schools of Dentistry and Medicine. University of Granada, Spain

## Abstract

**Background:**

The oral cavity is a reservoir for *Helicobacter pylori*, and occupations that involve close contact with it, like Dentistry, could entail a higher risk of colonization. The aim of this study was to evaluate if the length of stay at the School of Dentistry of the University of Granada could influence colonization by *H. pylori*, and to furthermore correlate colonization by *H. pylori*, with the state of oral health and oral hygiene habits.

**Material and Methods:**

The study groups were: Group 1, 35 students of Odontology in their first year of studies; Group 2, the same 35 students when they were in their fifth academic year; Group 3, 35 students from University study programs unrelated with Health and of the same age as the group 2 students. All underwent *H. pylori*, colonization determinations by means of the urea breath test (UBT), stool antigen test and a serological test. Also studied were the variables plaque index, gingival index and the number of times teeth were brushed per day. The Student t test was used for comparisons among the three studied groups. The Chi-squared test and Pearson correlation coefficient were used to determine any connection between colonization by *H. pylori*, and the variables studied.

**Results:**

Comparisons between groups 1 and 2 and between groups 2 and 3 showed significant differences regarding colonization by *H. pylori*, plaque index, gingival bleeding index and tooth brushing. A positive correlation was found between being colonized by *H. pylori*, and having a gingival index higher than 10% and tooth brushing once a day or less, in all the studied groups.

**Conclusions:**

Colonization by *H. pylori*, among Dentistry students at the University of Granada decreased over a four-year time period at the University. Factors related with better oral health, such as a lower gingival index and more frequent tooth brushings, would explain these results.

**Key words:**Dental students, Helicobacter pylori, serological test, stool antigen test, urea breath test.

## Introduction

*Helicobacter pylori* has been widely investigated since it was first isolated by Warren and Marshall in 1982. Related research efforts are fully justified given estimations that these bacteria colonize over half the world population, making this the most common chronic infection worldwide (1). This bacterial species is involved in many gastric and extra-gastric pathological disorders (1,2), and since 1994 it is moreover classified as a human carcinogen by the Working Group of the International Agency for Research on Cancer (2). In fact, gastric carcinoma, whose strongest known risk factor is *H. pylori*, is one of the most common malignancies globally and constitutes the second leading cause of cancer mortality worldwide (2).

There are several aspects of the bacteria that remain unclear, one being the way it is transmitted. While person-to-person appears to be the main means of transmission, the specific route -whether oral-oral, gastro-oral, or fecal-oral- has been widely debated (3).

The high prevalence of *H. pylori* reported in saliva, dental plaque and gingival sulcus has led researchers to consider the possibility that the oral cavity is the principal extragastric niche for *H. pylori* (3). A recent study shows that its presence in the oral cavity is significantly more prevalent among gastric *H. pylori*-positive patients than in gastric *H. pylori*-negative patients (4). Oral *H. pylori* also appears to be more difficult to eradicate than gastric *H. pylori*, possibly causing re-infection after a successful treatment (5). In addition, oral health is known to be related to the rates of infection healing and reduction of re-infection by this pathogen (6).

Since the oral cavity is a reservoir for *H. pylori*, occupations that involve close contact with it, including Dentistry, could be viewed as a risk factor for colonization. While performing dental procedures, aerosol sprays that can reach a distance of more than 1.5 m are created. Such aerosol spray may provide a transmission route for *H. pylori*, in the event it was carried by the patient, becoming a possible via of infection (7). However, the body of literature to date gives different prevalence results and contradictory conclusions (7-13). While Honda *et al.*(8) and Matsuda *et al.* (10) showed that dental professionals had a higher risk of infection than the general population, and Loster *et al.* (7) reported that the presence of *H. pylori* in the oral cavity of dentists appears to be related to the years of practice, other researchers such as Lin *et al.*(13) found no relationship between *H. pylori* and clinical dentistry. Just as dental professionals, dental students could constitute a population at risk because they perform clinical procedures involving contact with oral fluids. Thus, the longer their stay at the School of Dentistry, the greater the risk of colonization. However, few studies focus on this population (11-13), none of them are specific or recent and, what is more, all are cross-sectional studies relying on serological tests alone -without including more accurate techniques such as the urea breath test (UBT) and stool antigen test. The aim of this study was therefore to evaluate if a four-year stay at the School of Dentistry of the University of Granada influenced colonization by *H. pylori* as established using the UBT, stool antigen test and serological test, and to correlate *H. pylori* colonization with the state of oral health and oral hygiene habits.

## Material and Methods

- Study population

The study population comprised students of the University of Granada divided into three groups ( Table 1): Group 1, 1st-year students of the School of Dentistry that had not yet started their clinical practice; Group 2, the same students of the former group 1 who were recruited after 4 years of degree studies -including 3 years of clinical practice; and Group 3, a control group of students from different Schools outside the health sector who had no contact with dental patients. Groups 1 and 2 were included to evaluate colonization by *H. pylori* throughout their University training, and group 3 served to compare its colonization with that of group 2.

**Table 1:**
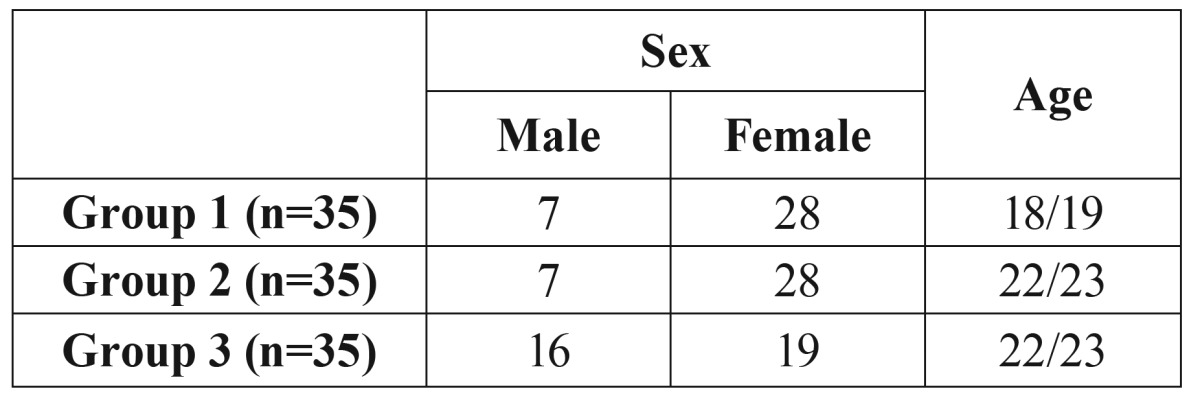
Sociodemographic data.

The exclusion criteria included having taken antisecretory agents, antibiotics, bismuth salts or corticosteroids within the previous two weeks. Students with extra-academic clinical activities or those who had been treated to eradicate *H. pylori* before or during the course of this study were also excluded.

The study was approved by the Ethics Committee of the School of Dentistry, and informed consent was obtained from all participating students before the study.

- Oral examination

An oral examination was performed on all the participants. The materials used were: a clinical exploration kit, plaque disclosing tablets, and other related disposable materials. The aim was to determine the plaque index (14) and gingival bleeding index (15). A 12 mg erythrosin pill (Plac-control®, Dentaid, Barcelona, Spain) was given to the students, who kept it in their mouths for 3 minutes, in order to calculate the plaque index. The cutoff point was established at 25%, meaning plaque values above 25% were classified as “poor brushing technique” and below it, as “good technique” (16). The gingival bleeding index was based on bleeding upon probing, the cutoff being set at 10%; values above it were considered “moderate to severe bleeding”, and below it, “lack of bleeding to mild bleeding” (16).

All examinations were performed by two dentists. The agreement of the results was evaluated by means of the Kappa index, giving results higher than 0.8, which indicated that it was “almost perfect”.

The subjects were also asked about how many times a day they brushed their teeth. This self-reported information was coded as brushing ≤ 1 per day or ≥ 2 per day.

- Determination of colonization by *H. pylori*

Colonization by *H. pylori* was determined using three different tests.

1. The Urea Breath Test (ref. 918 615, Otsuka Pharmaceuticals S.A., Barcelona, Spain) was used following the protocol recommended by the manufacturer. The students came for the breath test in the morning, after fasting for at least 8 hours. They were asked to rest for ten minutes before performing the test and to remain seated during it. The procedure consisted of taking two samples of expired air that were collected into bags, one before and one 20 minutes after ingesting a tablet of labeled urea with a solution of 4.2 g citric acid dissolved in water. After ingestion, the students rinsed their mouths with water to remove any residual labeled urea that could lead to false positives.

The final processing of the samples was performed using mass-spectrometry (Otsuka Pharmaceuticals S.A.). The result was expressed in delta units, representing the ratio 13CO2 / 12CO2 per thousand in relation to reference values. The levels of 13CO2 and 12CO2 were measured within the exhaled air prior to and after the ingestion of the labeled urea, the final result being the difference between the two measurements. Above 5 delta units is considered a positive value.

2. Stool Antigen Test: The immunochromatographic Letitest *H. pylori* Card (ref. PG820127F, Leti, Madrid, Spain,) was performed for the qualitative detection of *H. pylori* in stool, and was used following the manufacturer’s instructions; upon receipt of the sample, it was processed accordingly.

3. Serological Test: 5 mL of vein blood was obtained from every subject. The blood was centrifuged for 10 min at 2000 g and serum was saved for further investigation. Sera were tested by means of the Helicogen latex agglutination technique, which determines total antibodies against *H. pylori* (ref 3800-2603, Biokit S.A., Barcelona, Spain).

Based on the results obtained, the student was considered to be colonized by *H. pylori* if at least two of the three test results gave positive (8).

- Statistical analysis

All analyses were conducted with SPSS 21.0 version (IBM, Armonk, New York, USA). The Student t test was used for comparisons among the three studied groups, whereas the Chi-squared test and the Pearson correlation coefficient were used to identify associations between colonization by *H. pylori* and the variables of study (sex and variables related with the state of oral health and tooth brushing habits) in each one of the groups. The value of *p* < 0.05 was considered statistically significant.

## Results

 Table 2 shows the distribution of the students according to the studied variables (*H. pylori* colonization, plaque index, gingival bleeding index, sex and tooth brushing) as well as the comparisons between groups 1 and 2, and groups 2 and 3. Comparison of students in their first and in their last year of degree studies in Dentistry (groups 1 and 2) showed significant differences for colonization by *H. pylori*, plaque index, gingival bleeding index and tooth brushing. Similar differences were found when comparing groups 2 and 3, that is, 5th-year students of Dentistry with the control group, the variable sex also being statistically significant.

**Table 2:**
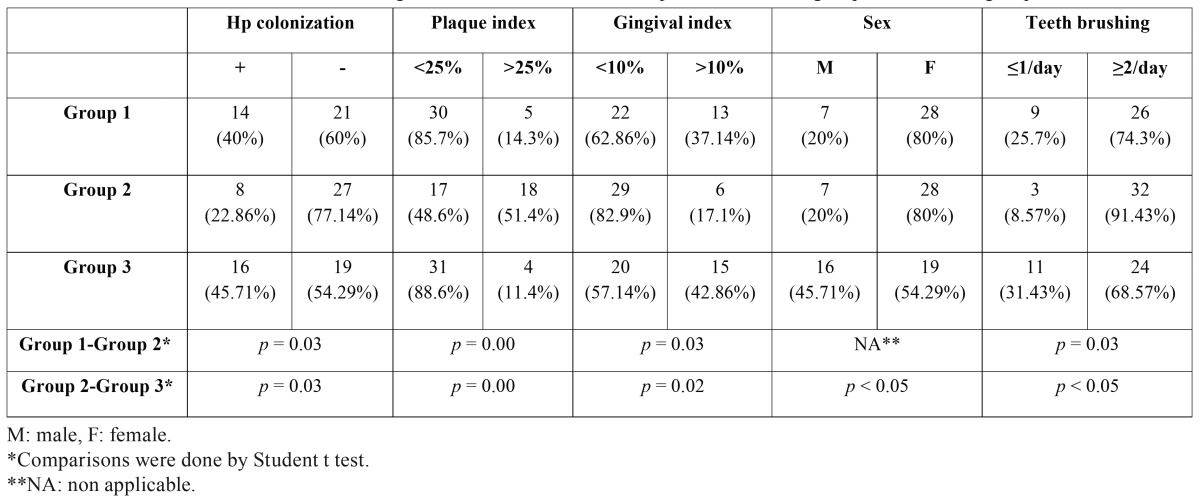
Distribution of the students according to the studied variables. Comparisons between groups 1 and 2, and groups 2 and 3.

In  Table 3 the correlation between *H. pylori* colonization and the variables in each group is shown. A positive correlation between the presence of *H. pylori* and gingival bleeding index and tooth brushing was found in all groups. Accordingly, *H. pylori* colonization was related to gingival bleeding indexes >10% and brushing teeth ≤ 1 a day. A positive correlation with sex was also found in group 2, in the sense that 5th-year Dentistry students colonized by *H. pylori* were usually men. No correlation was found with the plaque index in any of the groups.

**Table 3:**
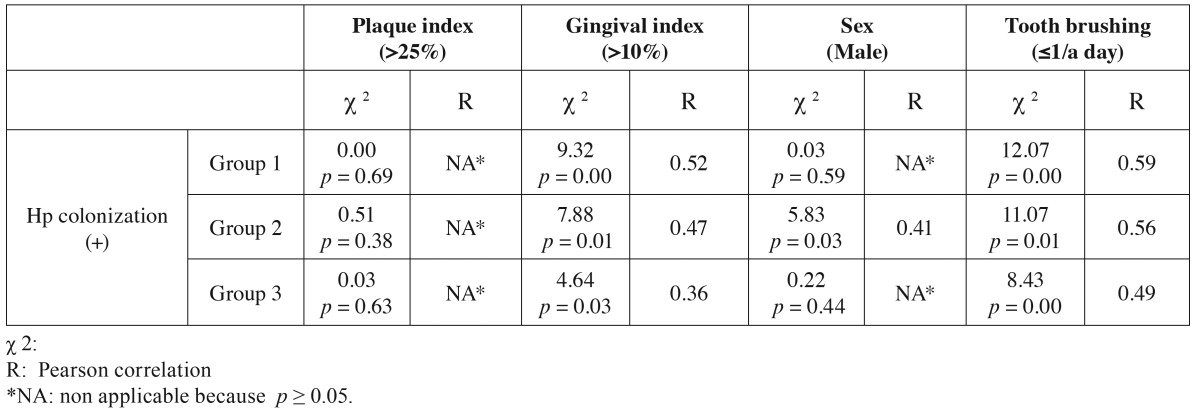
Correlation between Hp colonization and the studied variables in each one of the groups.

The strength of the correlation is expressed by the Pearson absolute value. In most cases correlations were moderate (0.40-0.69), except for the gingival bleeding index in group 3, whose correlation with colonization by *H. pylori* was low (0.2-0.39).

## Discussion

The frequent presence of *H. pylori* in the oral cavity led us to postulate that it might be considered a permanent reserve for this bacterial species. In such an event, working as a Dentist would be a risk factor for *H. pylori* colonization due to the emission of aerosol sprays during dental procedures (7). Nonetheless, the results of this study involving Dentistry students point in the other direction: we found a decreased presence of *H. pylori* colonization when comparing 5th-year students to students in their 1st year of studies. Meanwhile, a correlation was found between colonization by *H. pylori* and poor dental hygiene, represented by higher gingival indexes and a lesser number of tooth brushings per day.

Despite previous studies of professional dentists given their potentially higher risk of exposure to *H. pylori* (7-10), few studies have focused on Dentistry students (11-13). We chose to observe differences between students in their first and fifth (last) year of studies, furthermore examining the very same students over time, thus reducing confounding factors. A control group was additionally studied for comparison with the 5th-year Dentistry students under the premise that 1st-year students would present no different traits than the general population.

In order to determine colonization by *H. pylori*, three different diagnostic tests were applied. Previous studies recommend the use of at least two different tests to enhance sensitivity (17). Although endoscopy followed by biopsy is classically held as the gold standard for the diagnosis of infection-colonization by *H. pylori* (18), non-invasive techniques can be seen as a good alternative in the clinical practice, as they are cheaper and easier to perform, as well as accurate. At any rate, the use of a technique more invasive than serology would have exceeded the ethical limits of this research study.

Serology has an important limitation in that the presence of antibodies is not necessarily related with current infection-colonization, but it has the advantage of not being conditioned by local changes in the stomach that might lead to a decrease in the bacterial load and, therefore, a false negative (19). In the context of the present study, the serological assay was an agglutination in latex that detected total antibodies. Of all the methods available, the one of choice for detecting IgG antibodies is ELISA (19), but several studies report values for sensitivity and specificity that are comparable when detecting total antibodies (20,21).

For the detection of the antigen in feces we used a rapid immunochromatographic test. These assays are widely used, providing rates of sensitivity and specificity that are similar to those of other diagnostic techniques (22). The UBT was also performed, this being an optimal non-invasive means of detecting *H. pylori*, given its great diagnostic accuracy and the ease of its application (19).

The findings reported here show a decrease in *H. pylori* colonization over time among the Dentistry students studied. Indeed, *H. pylori* colonization is lower in the 5th-year students than among the general, non-clinical population represented by the control group. These results are in line with those of previous studies showing that studying dentistry was not a risk factor for infection/colonization by *H. pylori*. In fact, Lin *et al.*(13) and Malaty *et al.* (11) report lower seroprevalence data against *H. pylori* in the dental workers than in general population, while Banatvala *et al.* (12) found that seroprevalence was lower in clinical dental students than in pre-clinical dental students. It should be noted that in none of the cases the results were significant; however, these studies were based only on serology tests, they did not focus exclusively on dental students, and did not monitor participants throughout their studies.

The reason behind this decrease in oral colonization by *H. pylori* may lie in the improved dental hygiene of the students over time. The 5th-year Dentistry students showed better values for the gingival index and a greater number of tooth brushings per day. Numerous authors have arrived at associations between *H. pylori* colonization and oral health: Jia *et al.* (23) determined that dental plaque removal could help prevent infection or reinfection by *H. pylori*, comparing its prevalence, as determined by UBT, in subjects with and without plaque control; Sheu *et al.* (24) related the presence of dental disease with the recurrence of *H. pylori* infection. The findings by Andersen *et al.* (25) justify that dental plaque may serve as a reservoir for *H. pylori* given the ability of this bacteria to coaggregate with Fusobacterium nucleatum and Fusobacterium periodonticum, which are early and late colonizers, respectively, of gingival sulcus. In turn, Silva *et al.* (26) related colonization by *H. pylori* to the presence of plaque and gingival bleeding and Nouraie *et al.* to poor tooth brushing (27).

In our study, better results for the gingival index and tooth brushing were found for the 5th-year students with respect to the 1st-year students. Likewise, they gave far better results than the control group. These findings could be attributed to the more acute conscientiousness of Dental students over the course of their study program (28).

Even though we might have also anticipated lower levels of plaque among the 5th-year dental students, our results were paradoxically distributed. The participants presenting the best gingival indexes and tooth brushing habits (group 2) obtained the worst results for plaque indexes, and vice versa, the participants with the best plaque indexes (group 3) were the ones that brushed their teeth less and had worse gingival indexes. In group 1 the distribution was similar to that of the control group. This might be explained by the fact that the O´Leary index measures only the amount of plaque, whereas the gingival index measures the severity of the inflammatory response of the soft tissue around the teeth in the presence of plaque -that is, the long-term effects of plaque-thus providing more accurate information about the real state of oral health (29).

Because our study methodology did not involve bitonal plaque revealers, which could have allowed us to distinguish old plaque from recent plaque, we cannot ensure that the results obtained in the different groups actually reflect oral hygiene habits, rather than the result of brushing just before the oral examination. Indeed, it is logical that 5th-year Dentistry students would be well aware of their dental hygiene and hardly intimidated by the prospect of undergoing an oral exploration, whereas the control group could have experienced more anxiety and might have modified their behavior somewhat. This possibility introduces a factor of uncertainty of substantial importance when assessing the plaque index results.

Among the limitations of this study we acknowledge the different gender distribution of groups 1 and 2 with respect to group 3 as a remarkable one. However, it reflects a characteristic gender distribution in health and non-health degrees in our area. Gender could indeed be a confounding factor here if an unequal distribution of dental health habits and oral health, according to gender, could be demonstrated in our environment. Yet it would not affect the correlation between the presence of *H. pylori* and oral health established in the current study.

Our results show that the colonization by *H. pylori* among Dentistry students at the University of Granada was seen to decrease over the four-year period of study. Factors related with better oral health, such as a lower gingival index and more frequent tooth brushings, could explain them. It is important to highlight that the current study is the first to specifically evaluate the colonization of *H. pylori* in dental students throughout their university education using accurate diagnostic methods; our conclusion is that studying Dentistry per se cannot be considered a risk factor for the acquisition of *H. pylori*, but improving dental hygiene habits could be considered a protective factor for dental students. Still, further studies with a larger sample size and greater homogeneity of the study populations are needed to confirm these findings.
